# Prevalence and factors associated with the use of benzodiazepines among teachers in Espírito Santo: a cross-sectional study

**DOI:** 10.1590/1980-549720250014

**Published:** 2025-04-07

**Authors:** Yohan Cancilheri Mazzini, Bruna Lopes Antonucci, Guilherme Rocha Pereira, Walleri Christini Torelli Reis, Kérilin Stancine Santos Rocha, André Faro, Dyego Carlos Souza Anacleto de Araújo

**Affiliations:** I Universidade Federal do Espírito Santo - Vitória (ES), Brazil.; II Universidade Federal da Paraíba - João Pessoa (PB), Brazil.; III Universidade Federal de Sergipe - São Cristóvão (SE), Brazil.

**Keywords:** Faculty, Mental health, Psychotropic drugs, Anti-anxiety agents

## Abstract

**Objective:**

This study analyzed the prevalence and associated factors of benzodiazepine use among teachers in municipalities of Espírito Santo, Brazil, in 2024.

**Methods:**

A cross-sectional, quantitative study conducted with teachers from the state of Espírito Santo using self-administered questionnaires.

**Results:**

Among the 453 teachers surveyed, the prevalence of benzodiazepine use was 6.8% (n=31), increasing to 21.1% (n=26) among teachers with a previous diagnosis of mental disorders (n=123). Most benzodiazepines were used daily (57.6%; n=19), 42.5% (n=14) had been used for 2-5 years, and 39.4% (n=13) reported use without antidepressant treatment.

**Conclusions:**

Severe anxiety symptoms and clinical insomnia were associated with an increased likelihood of benzodiazepine use, whereas having a partner was associated with reduced use. The prevalence of benzodiazepine use among teachers was higher than in the general population.

## INTRODUCTION

Teachers are among the professionals most affected by stress, anxiety, depression, and sleep disturbances, often linked to psychosocial occupational risks. These risks include long working hours, multiple job commitments, pressure to achieve results, low perceived social support, exposure to violence, and salary dissatisfaction^1^. Such conditions may contribute to the use of benzodiazepines, GABA receptor agonists prescribed for anxiety and insomnia. However, chronic use of these medications can lead to tolerance and dependence^2^.

In Brazil, the illness and medicalization of teaching have garnered increasing research interest in recent years. However, most studies rely on small samples and convenience sampling. Furthermore, scientific publications primarily focus on antidepressant use, while knowledge regarding benzodiazepine use in this population remains limited^3^. In this context, the present study aimed to assess the prevalence of benzodiazepine use among teachers in Espírito Santo and identify associated factors.

## METHODS

This cross-sectional, analytical epidemiological study is part of the Teacher’s Mental Health research project, conducted in state schools in the cities of Vitória, Serra, Santa Teresa, and Fundão (Espírito Santo, Brazil). The study was approved by the Ethics Committee for Research with Human Beings (CAAE: 70203023.4.0000.5060).

A sample of 224 teachers was determined based on a finite population (n=2,662), with a 99% confidence level, a 5% margin of error, and an estimated outcome proportion of 3.6%, plus an additional 20% to account for losses and deff=2^4^. Single-stage cluster sampling was employed, with 20 of the 65 state schools randomly selected.

Data collection was conducted in person between January and February 2024 during the pedagogical planning day. The questionnaire included the following sections: sociodemographic profile; self-reported previous diagnosis of mental disorders; use of psychoactive medications; and screening scales for symptoms of anxiety (Generalized Anxiety Disorder - GAD-7), depression (Patient Health Questionnaire - PHQ-9), and insomnia (Insomnia Severity Index - IGI-2). These scales were cross-culturally adapted and validated for use in Brazil. The categorization of the variables used is detailed in the supplementary material.

A binary logistic regression analysis was conducted, adjusting for predictive variables of benzodiazepine use with p<0.20 in the unadjusted analysis. Odds ratios and 95% confidence intervals were estimated.

## RESULTS

A total of 453 teachers participated in the study, the majority of whom were cisgender women (63.1%, n=286). The mean age was 42.2±10.4 years. A previous diagnosis of mental disorders was reported by 27.2% (n=123). Benzodiazepine use was reported by 6.8% (n=31) of teachers overall and by 21.1% (n=26) of those who had reported a previous diagnosis of mental disorders.

Thirty-three instances of benzodiazepine use involving six different drugs were identified among 31 teachers. The majority of benzodiazepines were used daily (57.6%, n=19), and 42.5% (n=14) had been used for two to five years. Clonazepam was the most commonly used drug (60.7%, n=20), and 39.4% (n=13) of benzodiazepines were taken without concurrent use of antidepressants. The benzodiazepine use profile is detailed in Table 1.

**Table 1:** Benzodiazepines used by teachers from the state education system of Espírito Santo participating in the Teacher’s Mental Health study, 2024 (n=33).

Characteristics	n	%
Drug		
Alprazolam	7	21.2
Clonazepam	20	60.7
Clobazam	1	3.0
Cloxazolam	1	3.0
Diazepam	3	9.1
Chlordiazepoxide	1	3.0
Prescribing medical specialty		
General practitioner	2	6.1
Psychiatrist	22	66.7
Other	1	3.0
Not prescribed	2	6.1
Not reported	6	18.2
Frequency of use		
Daily	19	57.6
As needed (SOS)	9	27.3
Not reported	5	15.1
Duration of use (years)		
Up to one	8	24.2
Between two and five	14	42.5
Between six and 10	1	3.0
More than 10	2	6.1
Not reported	8	24.2
Use of benzodiazepine		
Isolated use	7	21.2
Associated with other psychotropic medications*. but without antidepressants	6	18.2
Associated with antidepressants (SSRIs ou SNRIs)	18	54.5
Associated with antidepressants (tricyclics)	2	6.1

*methylphenidate (n=1); trazodone and zolpidem (n=1); cannabidiol (n=1); zolpidem (n=1); lurasidone and quetiapine (n=2); SSRIs: selective serotonin reuptake inhibitors; SNRIs: serotonin-norepinephrine reuptake inhibitors.

The adjusted logistic regression model [χ^2^(12)=26.657; p=0.009] accounted for 32.1% of the variability in benzodiazepine use (Nagelkerke’s R^2^=0.321). Among the predictors included in the adjusted model, the presence of severe anxiety symptoms and clinical insomnia increased the likelihood of benzodiazepine use, whereas having a partner was associated with lower odds (Table 2). The results of the logistic regressions are available in the supplementary material (https://drive.google.com/file/d/13xgvNGm41wko--FDsiToPy4pyLuxOS_h/view).

**Table 2:** Adjusted odds ratio (OR) with 95% confidence intervals (95%CI) for benzodiazepine use among state school teachers in Espírito Santo, 2024.

Variables	Adjusted odds ratio (95%CI)
Gender identity	
Cisgender man	Reference
Cisgender woman	2.21 (0.76-6.41)
Gender minority	0.00
Marital status	
Single	Reference
With a partner	0.40 (0.17-0.94)
Employment status	
Permanent	Reference
Temporary	0.47 (0.20-1.12)
School location	
Vitoria	Reference
Serra	2.38 (0.72-7.83)
Fundão	5.51 (0.81-37.27)
Santa Teresa	1.40 (0.22-9.09)
Job satisfaction	
Satisfied	Reference
Neutral	1.07 (0.37-3.14)
Dissatisfied	2.14 (0.66-6.90)
Severe anxiety symptoms	
No	Reference
Yes	4.59 (1.72-12.27)
Severe depression symptoms	
No	Reference
Yes	1.40 (0.43-4.60)
Clinical insomnia symptoms	
No	Reference
Yes	2.68 (1.05-6.85)

## DISCUSSION

This study suggests that teachers may be particularly vulnerable to benzodiazepine prescriptions, as the prevalence of use in this population was higher than that observed in the global population (3%), the Brazilian population (3.6%), and Brazilians with mental disorders (7.8%)^2^ ^,^ ^4^. This vulnerability may be associated with a lack of psychosocial support, excessive workload, and increasing performance pressure, factors that can contribute to emotional exhaustion and the development of mental disorders such as anxiety and insomnia^1^, which are linked to benzodiazepine use. Furthermore, the findings highlight a gap in comprehensive teacher health care, where pharmacological interventions are often the primary response to psychological distress.

In this study, benzodiazepine use among teachers is particularly concerning given that one-third of these medications are used without concomitant antidepressants, and more than half are used chronically. This pattern may reflect a treatment approach centered on the immediate relief of anxiety and insomnia symptoms, while teachers continue to face the same stressors that contribute to these conditions. Although benzodiazepines provide rapid symptom relief, antidepressants are generally preferred, as they target the underlying causes of these disorders.

Logistic regression analysis indicated that having a partner serves as a protective factor against benzodiazepine use, supporting existing evidence that the presence of a partner is associated with improved mental well-being throughout life^5^. In contrast, teachers with severe anxiety symptoms were nearly five times more likely to use benzodiazepines, while those with clinical insomnia had almost three times the likelihood. Chronic use of these medications is associated with an increased risk of cognitive decline, reduced psychomotor and processing speed, and physical and psychological dependence, all of which may negatively impact teaching performance^6^.

The findings highlight the need to prioritize addressing the structural factors contributing to teacher illness by promoting improvements in working conditions and reducing stressors within the school environment. These efforts should include reviewing organizational factors such as long working hours, performance pressure, and insufficient institutional support, all of which directly impact teachers’ mental health. Simultaneously, complementary interventions at the individual level should be considered, including psychological support programs that emphasize non-pharmacological strategies whenever possible.

It is important to acknowledge that data collection was based on self-reporting, which may introduce response bias. Additionally, the findings are specific to teachers in the Espírito Santo state school system and may not be generalizable to other regions.
