# Supplementary material for: Prevalence and factors associated with the use of benzodiazepines among teachers in Espírito Santo: a cross-sectional study
Source: Rev Bras Epidemiol. 2025 Apr 7;28:e250014. doi: 10.1590/1980-549720250014 (PMC11980820; doi:10.1590/1980-549720250014)
Supplement: Translation

# Translation: Prevalência e fatores associados ao uso de benzodiazepínicos entre professores do Espírito Santo: um estudo transversal

**DOI:** 10.1590/1980-549720250014.2

**Authors:** Yohan Cancilheri Mazzini, Bruna Lopes Antonucci, Guilherme Rocha Pereira, Walleri Christini Torelli Reis, Kérilin Stancine Santos Rocha, André Faro, Dyego Carlos Souza Anacleto de Araújo

**Affiliations:** I Universidade Federal do Espírito Santo - Vitória (ES), Brasil.; II Universidade Federal da Paraíba - João Pessoa (PB), Brasil.; III Universidade Federal de Sergipe - São Cristóvão (SE), Brasil.

**Keywords:** Docentes, Saúde mental, Psicotrópicos, Ansiolíticos

## Abstract

**Objetivo:**

Analisar a prevalência e os fatores associados ao uso de benzodiazepínicos entre professores de municípios do Espírito Santo, Brasil, no ano de 2024.

**Métodos:**

Estudo transversal, quantitativo, realizado com professores do estado do Espírito Santo por meio de questionários autoaplicados.

**Resultados:**

Entre os 453 professores, a prevalência de uso de benzodiazepínicos foi de 6,8% (n=31) e de 21,1% (n=26) entre professores com diagnóstico prévio de transtornos mentais (n=123). A maioria dos benzodiazepínicos é utilizada diariamente (57,6%; n=19), 42,5% (n=14) deles têm sido utilizados entre dois e cinco anos, e 39,4% (n=13) são utilizados na ausência de antidepressivos.

**Conclusões:**

Ter sintomas de ansiedade grave e insônia clínica aumenta as chances de uso de benzodiazepínicos, enquanto possuir companheiro(a) as reduz. O uso de benzodiazepínicos entre professores foi maior que entre a população em geral.

## INTRODUÇÃO

Os professores estão entre os profissionais mais afetados por estresse, ansiedade, depressão e problemas de sono, frequentemente relacionados aos riscos ocupacionais psicossociais. Esses riscos incluem longas jornadas de trabalho, múltiplos empregos, pressão por resultados, baixa percepção de apoio social, exposição à violência e insatisfação salarial^1^. Essas condições podem levar ao uso de benzodiazepínicos, agonistas gabaérgicos usados no tratamento de sintomas de ansiedade e insônia, no entanto o uso crônico desses medicamentos pode resultar em tolerância e dependência^2^.

No Brasil, o adoecimento e a medicalização do trabalho docente têm despertado crescente interesse entre pesquisadores nos últimos anos, porém a maioria dos estudos utiliza amostras pequenas e amostragem por conveniência. Além disso, publicações científicas focam no uso de antidepressivos, sendo limitado o conhecimento sobre o uso de benzodiazepínicos nessa população^3^. Nesse contexto, este estudo objetivou avaliar a prevalência e fatores associados ao uso de benzodiazepínicos entre professores do Espírito Santo.

## MÉTODOS

Trata-se de um estudo epidemiológico transversal e analítico que integra o projeto de pesquisa Saúde Mental do Professor, realizado nas escolas estaduais dos municípios de Vitória, Serra, Santa Teresa e Fundão (Espírito Santo, Brasil), aprovado pelo Comitê de Ética em Pesquisa com Seres Humanos (CAAE: 70203023.4.0000.5060).

Foi calculada uma amostra de 224 professores, com base em uma população finita (n=2.662), nível de confiança de 99%, erro de 5%, proporção estimada do desfecho de interesse de 3,6%, acrescida de 20% para perdas e *deff*=2^4^. Utilizou-se a amostragem por conglomerado em único estágio, sorteando-se 20 das 65 escolas estaduais.

A coleta de dados foi realizada presencialmente entre janeiro e fevereiro de 2024, durante a jornada de planejamento pedagógico. O questionário continha as seções: perfil sociodemográfico; autorrelato de diagnóstico prévio de transtornos mentais; uso de medicamentos psicoativos; escalas de rastreamento de sintomas de ansiedade (Generalized Anxiety Disorder-7; GAD-7), depressão (Patient Health Questionnaire-9; PHQ-9) e insônia (Índice de Gravidade de Insônia-2; IGI-2), adaptadas transculturalmente e com evidências de validade para uso no Brasil. A categorização das variáveis utilizadas está descrita no material suplementar. 

Foi realizada uma regressão logística binária, ajustada para as variáveis preditoras do uso de benzodiazepínicos com p<0,20 na análise não ajustada. Foram estimados os valores de *odds ratio* e intervalos de confiança de 95%.

## RESULTADOS

Participaram do estudo 453 professores, sendo a maioria mulheres cisgênero (63,1%, n=286). A média de idade foi de 42,2±10,4 anos. O diagnóstico prévio de transtornos mentais foi relatado por 27,2% (n=123). O uso de benzodiazepínicos foi relatado por 6,8% (n=31) dos professores em geral e por 21,1% (n=26) dos professores que haviam relatado diagnóstico prévio de transtornos mentais.

Foram identificadas 33 ocorrências de uso de seis fármacos benzodiazepínicos por 31 professores. A maioria dos benzodiazepínicos é utilizada diariamente (57,6%; n=19), e 42,5% (n=14) deles têm sido utilizados entre dois e cinco anos. O fármaco mais utilizado foi o clonazepam (60,7%; n=20), e 39,4% (n=13) dos benzodiazepínicos são utilizados na ausência de antidepressivos. O perfil de uso de benzodiazepínicos está descrito na Tabela 1.

**Tabela 1:** Benzodiazepínicos em uso por professores da rede estadual do Espírito Santo participantes do estudo Saúde Mental do Professor, 2024 (n=33).

Variáveis	n	%
Fármaco		
Alprazolam	7	21,2
Clonazepam	20	60,7
Clobazam	1	3,0
Cloxazolam	1	3,0
Diazepam	3	9,1
Clordiazepóxido	1	3,0
Especialidade médica prescritora		
Clínico geral	2	6,1
Psiquiatra	22	66,7
Outro	1	3,0
Não prescrito	2	6,1
Não relatou	6	18,2
Frequência de uso		
Diário	19	57,6
Quando necessário (SOS)	9	27,3
Não relatou	5	15,1
Tempo de uso (ano)		
Até um	8	24,2
Entre dois e cinco	14	42,5
Entre seis e 10	1	3,0
Acima de 10	2	6,1
Não relatou	8	24,2
Uso do benzodiazepínico		
Uso isolado	7	21,2
Uso associado a outros medicamentos psicotrópicos*, mas na ausência de antidepressivo	6	18,2
Associado a antidepressivo (ISRS ou IRSN)	18	54,5
Associado a antidepressivo (tricíclicos)	2	6,1

*metilfenidato (n=1); trazodona e zolpidem (n=1); canabidiol (n=1); zolpidem (n=1); lurasidona e quetiapina (n=2); ISRS: inibidor seletivo da recaptação de serotonina; IRSN: inibidor da recaptação de serotonina e noradrenalina.

O modelo de regressão logística ajustada [χ^2^(12)=26,657; p=0,009] foi capaz de explicar 32,1% (R^2^ de Nagelkerke=0,321) da variabilidade no uso de benzodiazepínicos. Entre os preditores incluídos no modelo ajustado, ter sintomas de ansiedade grave e insônia clínica aumenta as chances de uso de benzodiazepínicos, enquanto possuir companheiro(a) as reduz (Tabela 2). Os resultados das regressões logísticas estão disponíveis no material suplementar (https://drive.google.com/file/d/13xgvNGm41wko--FDsiToPy4pyLuxOS_h/view).

**Tabela 2:** *Odds ratio* (OR) ajustado com intervalos de confiança de 95% (IC95%) de uso de benzodiazepínicos entre professores da rede estadual de ensino do Espírito Santo, 2024.

Variáveis	Uso de benzodiazepínicos OR ajustado (IC95%)
Identidade de gênero	
Homem cisgênero	Referência
Mulher cisgênero	2,21 (0,76-6,41)
Minoria de gênero	0,00
Situação conjugal	
Solteiro(a)	Ref.
Possui parceiro(a)	0,40 (0,17-0,94)
Vínculo empregatício	
Efetivo	Ref.
Temporário	0,47 (0,20-1,12)
Localização das escolas	
Vitoria	Ref.
Serra	2,38 (0,72-7,83)
Fundão	5,51 (0,81-37,27)
Santa Teresa	1,40 (0,22-9,09)
Satisfação com o trabalho	
Satisfeito	Ref.
Neutro	1,07 (0,37-3,14)
Insatisfeito	2,14 (0,66-6,90)
Sintomas de ansiedade grave	
Não	Ref.
Sim	4,59 (1,72-12,27)
Sintomas de depressão grave	
Não	Ref.
Sim	1,40 (0,43-4,60)
Sintomas de insônia clínica	
Não	Ref.
Sim	2,68 (1,05-6,85)

## DISCUSSÃO

Este estudo sugere uma vulnerabilidade docente à prescrição dos benzodiazepínicos, visto que a prevalência do uso dessa medicação entre os professores foi maior que a prevalência observada na população mundial (3%), na população brasileira (3,6%) e entre brasileiros com transtornos mentais (7,8%)^2^ ^,^ ^4^. Essa vulnerabilidade pode estar relacionada à falta de suporte psicossocial, à sobrecarga de atividades e à crescente cobrança por desempenho, que podem levar ao esgotamento emocional e a transtornos mentais, como ansiedade e insônia^1^, os quais estão relacionados ao uso desses medicamentos. Além disso, revela uma lacuna no cuidado integral à saúde docente, que utiliza intervenções farmacológicas como resposta primária ao sofrimento psíquico.

Neste estudo, o uso de medicamentos por docentes se torna mais preocupante quando se considera que um terço dos benzodiazepínicos é empregado na ausência de antidepressivos e mais da metade é usado cronicamente. É possível que a prescrição reflita uma abordagem focada no alívio imediato dos sintomas de ansiedade e insônia, enquanto os professores permanecem submetidos aos mesmos agentes estressores que originam esses problemas. Embora os benzodiazepínicos ofereçam alívio imediato dos sintomas, os antidepressivos são preferidos por atuarem nas causas subjacentes dos transtornos.

A regressão logística indicou que possuir companheiro(a) é um fator protetivo para o uso de benzodiazepínicos, reforçando as evidências de que a presença de um parceiro está associada à melhora do bem-estar mental ao longo da vida^5^. Em contrapartida, professores com sintomas de ansiedade grave têm quase cinco vezes mais chances de utilizar benzodiazepínicos e aqueles com insônia clínica chance quase três vezes maior. O uso crônico desses medicamentos pode levar a maior risco de declínio cognitivo, redução da velocidade psicomotora e de processamento, dependência física e psicológica, com potencial para impactar negativamente no desempenho da docência^6^.

Os dados demonstram a necessidade de priorizar a abordagem dos fatores estruturais que contribuem para o adoecimento dos professores, promovendo mudanças nas condições de trabalho e a redução dos estressores presentes no contexto escolar. Essas mudanças incluem a revisão de fatores organizacionais, como as longas jornadas de trabalho, a pressão por resultados e a falta de apoio institucional, que impactam diretamente a saúde mental dos docentes. Paralelamente, é importante considerar intervenções complementares direcionadas aos indivíduos, como programas de suporte psicológico que priorizem estratégias não farmacológicas sempre que possível.

Importa destacar que a coleta de dados se baseou no autorrelato, o que pode gerar vieses de resposta. Além disso, os resultados são específicos para professores da rede estadual do Espírito Santo e podem não ser generalizáveis para outras regiões.
